# The association of Life’s Simple 7 and infertility among U.S. women

**DOI:** 10.3389/fendo.2024.1288289

**Published:** 2024-02-01

**Authors:** Lixia Wang, Guangting Chang, Shu Cai, Xiaofang Zou, Meijiao Qin, Yingyao Tan

**Affiliations:** ^1^ School of Nursing, Guangdong Pharmaceutical University, Guangzhou, China; ^2^ Department of Nursing, The Third Affiliated Hospital of Guangzhou Medical University, Guangzhou, China; ^3^ Department of Nursing, Shenzhen Longgang District Maternal and Child Health Hospital, Shenzhen, China

**Keywords:** infertility, cardiovascular health (CVH), Life’s Simple 7 (LS7), National Health and Nutrition Examination Survey (NHANES), female

## Abstract

**Background:**

The Life’s Simple 7 (LS7) metric is a comprehensive measure of cardiovascular health (CVH) that encompasses seven distinct risk factors and behaviors associated with cardiovascular disease (CVD). Some studies have shown an association between infertility and CVD. The present study aimed to explore the potential association between the LS7 factors and infertility.

**Methods:**

A cross-sectional study was conducted on a sample of 3537 women aged 18-44 years from the National Health and Nutrition Examination Survey (NHANES) spanning the years 2013-2018. The LS7 metrics encompassed various factors including physical activity, smoking habits, body mass index, blood pressure levels, dietary patterns, blood glucose levels, and total cholesterol levels. We computed a 14-point LS7 score based on participants’ baseline data, classifying them as “inadequate” (3-6), “average” (7-10), or “ideal” (11-14). Infertility is defined as an affirmative answer to either of two questions on the NHANES questionnaire: “Have you tried to conceive for at least one year without success?” and “Have you sought medical help for your inability to conceive?” Logistic regression was utilized to estimate odds ratios (O.R.s) and 95% confidence intervals (C.I.s).

**Results:**

In total, 17.66% of participants were classified as individuals who reported experiencing infertility. In the continuous analysis, each one-unit increase in LS7 score was associated with a significantly decreased odds of infertility (OR=0.88 [0.77-0.89]). Analyzing the categorical representation of LS7 score, compared to individuals with poor scores, those with ideal scores exhibited a substantial 58% reduction in the odds of infertility (OR=0.42 [0.26-0.69]). Additionally, the observed interaction suggested that the influence of age on the relationship between LS7 and infertility is not consistent across different age groups (*P* for interaction < 0.001). Among individuals aged 35 or younger, each unit increase in LS7 score was associated with a substantial 18% (OR=0.82 [0.76-0.89]) decrease in the odds of infertility. However, in the older age group (>35), the association was attenuated and non-significant.

**Conclusions:**

Our research suggests a significant inverse association between LS7 scores and infertility. Age demonstrated a varying impact on this relationship, with a more pronounced impact observed among individuals aged 35 or younger.

## 1 Introduction

Infertility is a medical condition that has traditionally been defined as the incapacity to achieve a viable pregnancy following a period of 12 months or longer of regular, unprotected sexual intercourse. It is estimated to impact approximately 8.5% of women in the United States between the ages of 15 and 49 (1, 2). Infertility, as highlighted by the Centers for Disease Control and Prevention (CDC) in the United States, extends beyond a mere quality-of-life concern, encompassing substantial public health implications including psychological distress, societal marginalization, financial strain, and marital disharmony (3). Moreover, it should be noted that infertility is not exclusively a distinct ailment of the reproductive system, but rather frequently exhibits physiological or genetic associations with various other diseases and conditions, including cardiovascular disease (4, 5).

Cardiovascular disease (CVD) stands as a prominent contributor to mortality rates within the United States (6). Polycystic ovary syndrome (PCOS), a well-established factor contributing to infertility, has been linked to compromised glucose tolerance and cardiovascular disorders (7, 8). Approximately 25% of cases of female-factor infertility are attributed to anovulation related to PCOS, while a significant proportion remains unexplained. The National Health and Nutrition Examination Survey (NHANES) has found a significant association between the experience of infertility in women and cardiovascular health (9). Nevertheless, the correlation between cardiovascular health metrics and infertility lacks sufficient evidence, making it necessary to investigate further in order to elucidate the impact of cardiovascular disease prevention on women experiencing infertility.

The American Heart Association (AHA) has developed a health guideline known as Life’s Simple 7 (LS7), which serves as a metric for cardiovascular (CV) health (10). LS7 categorizes individuals into poor, intermediate, and ideal levels based on seven CVD risk factors and behaviors: smoking, physical activity, body mass index (BMI), total cholesterol, fasting glucose, blood pressure, and diet (11). Multiple studies have demonstrated a correlation between elevated LS7 scores, which serve as a measure of ideal cardiovascular well-being, and a lower risk of developing cardiovascular disease (12) and non-cardiovascular disease outcomes such as heart failure (13, 14), cancer (15), depression, and cognitive impairment (16), To date, there has been a dearth of research investigating the potential association between Ideal cardiovascular health and infertility.

As a result, the present study investigates the association between LS7 and infertility in women. The study utilizes a substantial sample size comprising individuals aged 18 to 44, sourced from the National Health and Nutrition Examination Study (NHANES).

## 2 Methods

### 2.1 Study population

The NHANES is a comprehensive, multistage, and probabilistic survey of the U.S. national population that offers a wealth of data on the general health and nutrition of the U.S. population (17). The NHANES employs a meticulously structured stratified, multistage probability sampling design to systematically enlist a representative cohort from the U.S. civilian, non-institutionalized populace. This sampling strategy entails the meticulous division of the nation into diverse strata based on nuanced demographic and geographic characteristics. Within each stratum, discrete clusters are identified, and a randomized selection of households ensues. Selected participants undergo a comprehensive health examination encompassing meticulous medical, dental, and physiological assessments. Simultaneously, participants partake in detailed interviews to elicit comprehensive information on various health-related domains, encompassing demographics, lifestyle patterns, and dietary habits. Furthermore, the research protocol includes the collection of biological specimens, such as blood and urine, facilitating the assessment of health indicators, nutritional status, and the determination of exposure levels to environmental factors.

**Figure 1:**
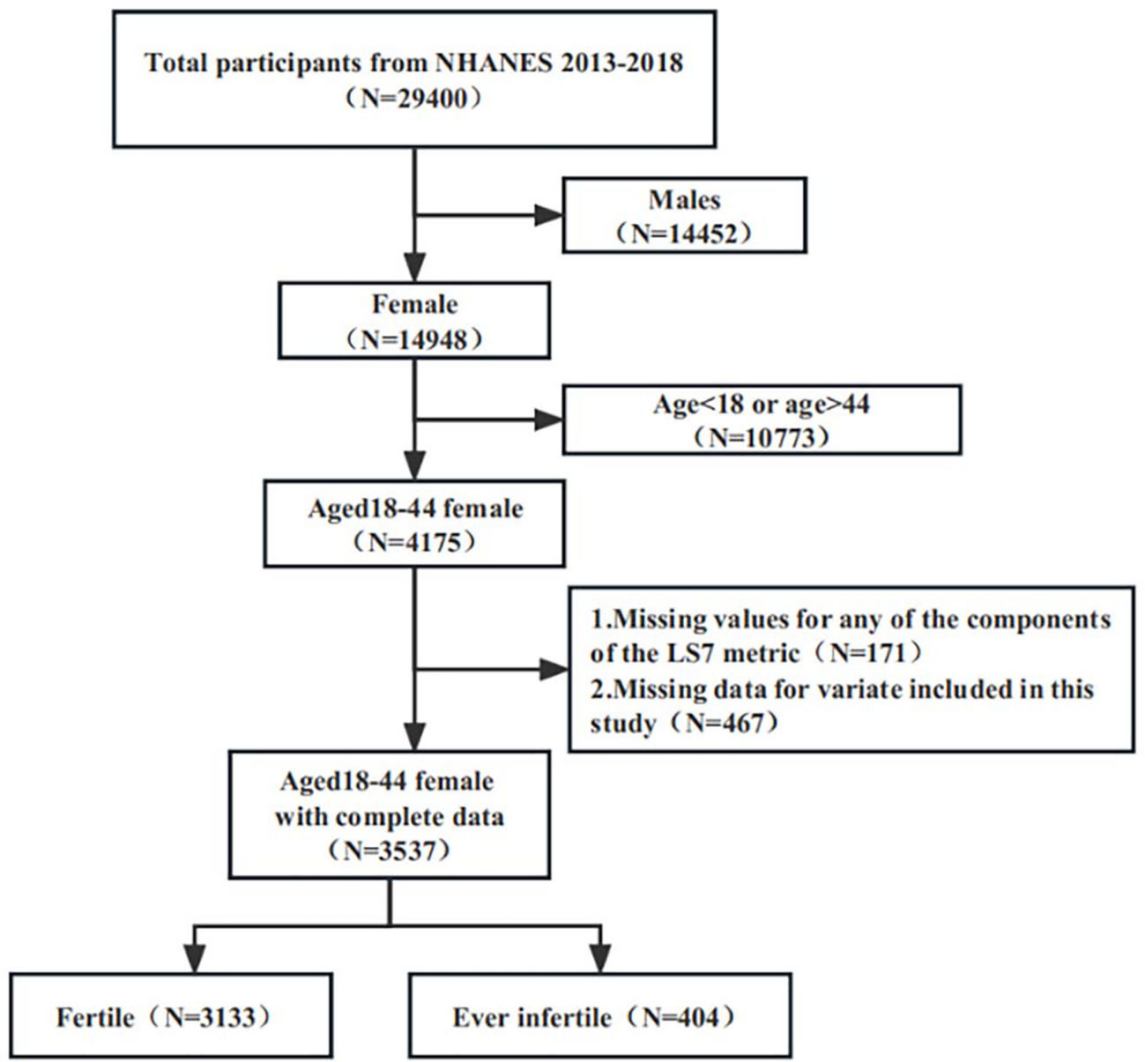
Flowchart of participant selection. NHANES, National Health and Nutrition Examination Survey.

The 2013–2018 continuous cycle of the US NHANES dataset was used for this investigation. A total of 29,400 individuals took part in the three cycles under consideration. After excluding male participants (N=14452), individuals below the age of 18 or above the age of 44 (N=10733), those with any missing data for the LS7 metrics (N=171), and individuals with missing data for any of the variables included in this study (N=467), a total of 3537 participants were included for analysis (see **Figure 1**).

### 2.2 Study variables

Information about age, marital status, education, race and ethnicity, poverty level index and smoking status were all self-reported. The measurement of socioeconomic status involved the utilization of the poverty income ratio, which represents the proportion of family income in relation to the federal poverty threshold, taking into account the specific year in which the interview was conducted. Poverty was operationally defined as a ratio equal to or less than one (18).

The LS7 framework encompasses various factors that contribute to overall health, including physical activity, smoking habits, BMI, dietary patterns, blood glucose levels, blood pressure, and total cholesterol levels. The measurement of height and weight was conducted using standardized methodologies. BMI was calculated as weight in kilograms over height in meters squared (kg/m²), and was categorized using criteria established by the National Institutes of Health as underweight (<18.5 kg/m²), normal (18.5–24.9 kg/m²), overweight (25.0–29.9 kg/m2), and obese (≥30 kg/m2). Due to the relatively small number of respondents in the underweight category, the underweight category was joined with the normal category after a sensitivity analysis showed little difference in the results between excluding the underweight category and including them in the normal weight category (19). Specifically, to measure height accurately, one should remove shoes and bulky clothing, use a flat headpiece to form a right angle with the wall, and measure from the base on the floor to the marked measurement on the wall using a metal tape. To measure weight accurately, one should use a digital scale, avoid using bathroom scales that are spring-loaded, and ensure that the scale rests on a firm, stable table. The assessment of physical activity involved quantifying the frequency of engaging in activities of moderate to vigorous intensity, such as walking, jogging, running, bicycling, swimming, dancing, or yard work, within the preceding 30-day period. The levels of glucose and total cholesterol were assessed using previously established methodologies (20). The assessment of diet intake was conducted by interviewers who had received specialized training. This assessment involved the use of two 24-hour dietary recall questionnaires. The U.S. Department of Agriculture utilized the mean of two recalls pertaining to various dietary components (such as fruits and vegetables, fish, whole grain, sodium, and added sugar) in order to calculate the healthy diet index. The risk factors associated with cardiovascular disease were classified into three levels: “poor,” “intermediate,” and “ideal.” Each level was assigned a score of 0, 1, and 2, respectively (refer to **Appendix 1**). The scores were aggregated, with a maximum value of 14 representing the ideal level of cardiovascular health. The LS7 lacks validated cut points, and the cut points employed in previous studies have not demonstrated consistency. Nevertheless, numerous studies have demonstrated that individuals with scores of 10 or 11 or higher exhibit a reduced occurrence of both cardiovascular and non-cardiovascular diseases (21, 22). In accordance with earlier studies, the entire LS7 score was therefore categorized as being insufficient (0–7), average (8-10), or ideal (11–14) (23–25).

Infertility was assessed by each woman’s response to two questions from the NHANES questionnaire: 1) “Have you ever attempted to become pregnant over a period of at least a year without becoming pregnant?” and 2) “Have you ever been to a doctor or other medical provider because you have been unable to become pregnant?” Any woman who answered “Yes” to either of these questions was considered to have a history of infertility.

### 2.3 Statistical analysis

The statistical analysis was conducted utilizing the statistical computing and graphics software R (version 4.3.1). The baseline characteristics of the participants were presented using mean values with standard error (SE) and proportions. Categorical variables were analyzed using the Rao-Scott x2 test, while continuous data were analyzed using analysis of variance. Logistic regression models were employed, taking into account the weighting of the data. The study employed logistic regression models to calculate odds ratios (O.R.s) and 95% confidence intervals (C.I.s) in order to assess the relationship between ideal cardiovascular health, represented as continuous or categorical variables, and fertility status. The Benjamini-Hochberg (BH) method was used to control the false discovery rate (FDR) for multiple testing. The multivariate test was constructed utilizing three models. Model 1 did not include any adjusted variables. In Model 2, adjustments were made for age, race, and education. Model 3 included adjustments for age, race and ethnicity, education, marital status, education, and poverty level index. The NHANES study has previously established associations between sociodemographic characteristics and infertility (26, 27). No additional adjustments were made for clinical parameters, including diabetes, hypertension, obesity, dyslipidemia, or hypertension. This occurred due to the measurements already accounted for in the estimation of the LS7 scores. Subsequently, the aforementioned statistical study methodologies were implemented for the subgroups pertaining to age, poverty level index, and marital status. Statistical significance was established at a significance level of P<0.05. Weighting approach was used to make the results more reflective of the broader US population.

## 3 Results

### 3.1 Baseline characteristics

**Figure 1** depicts the study participants’ selection process. Following selection, 3537 suitable participants were included for analysis, reflecting a population of 50,982,232 in the United States. 3133 people (82.34%, representing a population of 44,528,032) were fertile, while 404 (17.66%, representing a population of 6,454,200) were ever infertile.

**Table 1** presents the differences in the chosen participants’ baseline characteristics. Significant differences were observed between the fertile and infertile groups in the context of weighted analyses. The ever-infertile group differs from the fertile group in that they are more likely to be older (34.54 years vs. 30.38 years, *P*<0.001), to be married (63.81% vs. 41.71%, *P*< 0.001), and to have lower scores for smoking, blood pressure, body mass index, and glucose in the estimation of the LS7 scores. However, they have a lower proportion of poverty level index (<=1.3). All in all, Infertile individuals had a lower mean LS7 score than those in the fertile group (8.80 ± 0.18 vs 9.52 ± 0.07, *P*<0.001).

**Table 1:** Weighted characteristics of the study population based on selected participants (weighted sample, N= 50,982,232).

Characteristics	Fertile	Ever infertile	*P-value*
	N= 3133	N= 404	
Age (years)	30.38 ± 0.21	34.54 ± 0.50	<0.001
Education lever (%)			0.461
More than high school	65.61	69.32	
High school	21.23	19.87	
Less than high school	13.16	10.80	
Race/Ethnicity (%)			0.130
Mexican American	12.39	10.63	
Non-Hispanic Black	13.64	12.48	
Non-Hispanic White	54.63	61.58	
Other Hispanic	8.26	5.98	
Other Race	11.09	9.34	
Poverty level index (%)			0.007
<=1.3	30.81	24.97	
1.3-1.85	12.08	7.89	
>1.85	57.10	67.14	
Marital status (%)			<0.001
Married	41.71	63.81	
Divorced/Separated/Widowed	10.15	11.24	
Never married	33.27	13.97	
Living with partner	14.87	10.98	
Life’s Simple 7 components			
Physical activity score	1.82 ± 0.01	1.82 ± 0.03	0.980
Smoking score	1.52 ± 0.02	1.40 ± 0.05	0.033
Blood pressure score	1.72 ± 0.01	1.62 ± 0.03	0.019
Body mass index score	1.01 ± 0.03	0.77 ± 0.07	0.003
Glucose score	1.84 ± 0.01	1.71 ± 0.03	<0.001
Cholesterol score	1.69 ± 0.01	1.56 ± 0.05	0.072
Dietary intake score	0.51 ± 0.02	0.50 ± 0.03	0.980
Life’s Simple 7 score	9.52 ± 0.07	8.80 ± 0.18	<0.001

Mean ± SE for continuous variables: P-value value was calculated by weighted analysis of variance.

Percentages for categorical variables: P-value was calculated by weighted chi-square test.

The Benjamini-Hochberg method was used to adjust p values for multiple testing.

**Table 2** presents a comprehensive compilation of the clinical characteristics exhibited by the subjects, with a particular focus on their cardiovascular health status, which is categorized as a column-stratified variable. When comparing the normal group to the group of participants classified as having ideal cardiovascular health, it was observed that a higher percentage of individuals in the latter group were younger (73.56% aged <=35 years), non-Hispanic white (57.55%), living above the poverty threshold (65.09%), and had education beyond high school (74.80%).

**Table 2:** Characteristics of the selected participants According to Cardiovascular Health Status.

Characteristics	Inadequate	Average	Ideal
Age (%)			
<=35	45.64	63.05	73.56
>35	54.36	36.95	26.44
Education lever (%)			
Less than high school	16.84	14.70	8.15
High school	26.24	21.86	17.04
More than high school	56.91	63.43	74.80
Race/Ethnicity (%)			
Mexican American	11.63	12.55	11.96
Non-Hispanic Black	19.94	14.02	9.15
Non-Hispanic White	53.16	54.97	57.55
Other Hispanic	6.27	7.77	9.20
Other Race	9.00	10.69	12.16
Poverty level index (%)			
<=1.3	37.31	31.99	23.35
1.3-1.85	14.24	10.36	11.55
>1.85	48.45	57.65	65.09
Marital status (%)			
Married	36.61	45.80	48.10
Divorced/Separated/Widowed	16.33	10.73	6.02
Never married	30.77	27.98	34.31
Living with partner	16.30	15.49	11.57
Life’s Simple 7 components			
Physical activity score (%)			
Poor	0	0	0
Intermediate	34.86	20.79	9.64
Ideal	65.14	79.21	90.36
Smoking score (%)			
Poor	42.64	21.15	2.13
Intermediate	15.10	12.40	8.77
Ideal	42.26	66.45	89.10
Blood pressure score (%)			
Poor	12.73	2.55	0.09
Intermediate	45.22	24.74	7.83
Ideal	42.05	72.71	92.08
Body mass index score (%)			
Poor	76.62	47.36	6.87
Intermediate	15.10	27.66	24.51
Ideal	8.27	24.97	68.62
Glucose score (%)			
Poor	13.44	1.08	0.00
Intermediate	30.42	11.93	2.21
Ideal	56.13	87.00	97.79
Cholesterol score (%)			
Poor	14.95	5.98	0.80
Intermediate	42.31	22.14	9.49
Ideal	42.75	71.88	89.72
Dietary intake score (%)			
Poor	76.10	59.86	29.72
Intermediate	22.82	39.63	63.21
Ideal	1.09	0.51	7.07
Ever infertile			
No	83.02	85.48	92.26
Yes	16.98	14.52	7.74

Life’s Simple 7 score (0-14) Inadequate: (0-7); Average: (8-10); Ideal: (11-14).

Differences in characteristics by cardiovascular health status were all statistically significant (P<0.05); Values are survey-weighted percentages.

**Figure 2:**
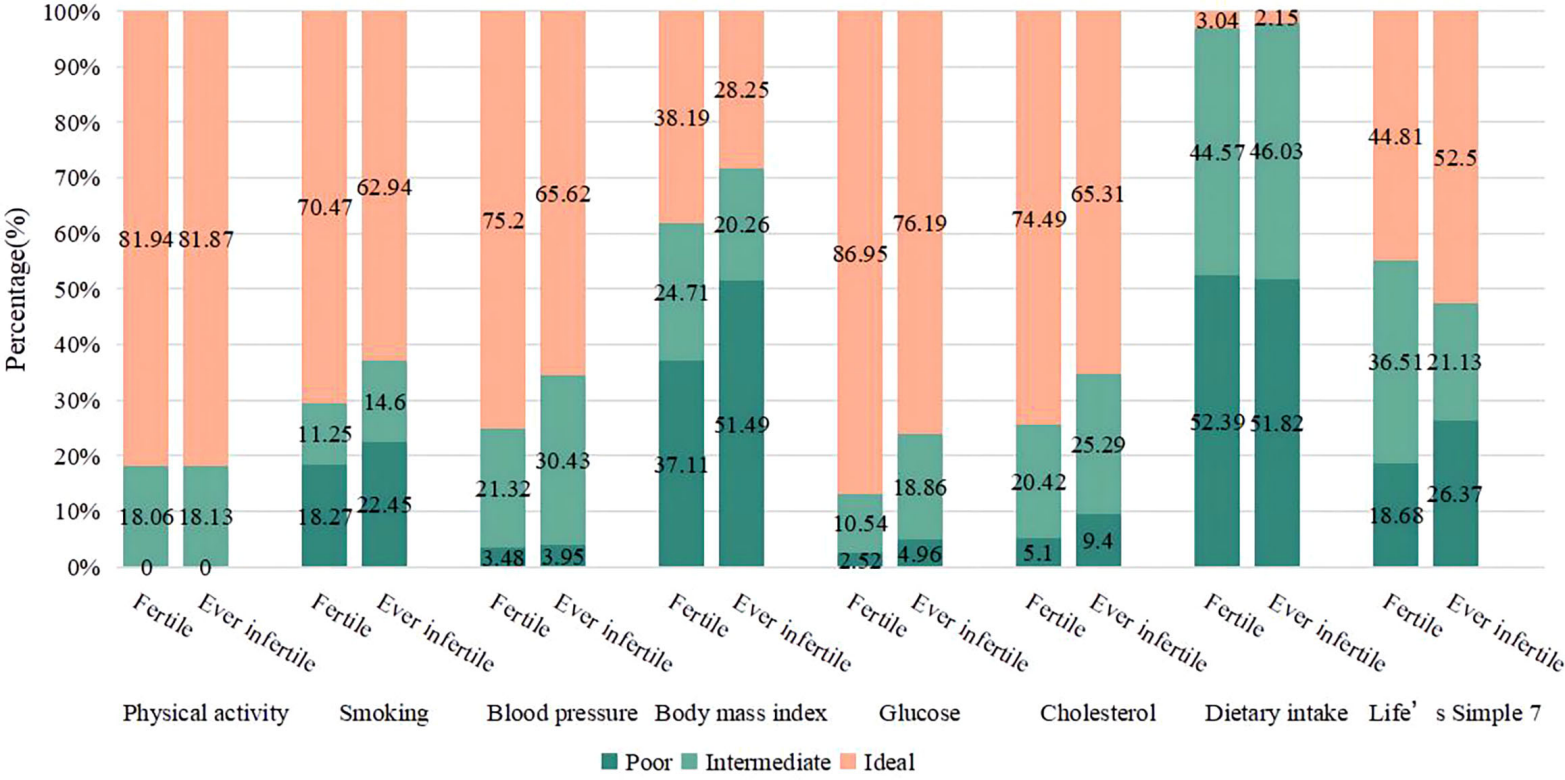
Distribution of Life’s Simple 7 components in fertile and ever infertile subjects. Differences in poor, intermediate and ideal groups by fertility status were all statistically significant (P<0.01). Life’s Simple 7 score Poor: (0); Intermediate: (1); Ideal: (2).

**Figure 2** displays the distribution of LS7 components among individuals classified as fertile and ever infertile. Among the fertile population, a significant proportion of participants performed well in terms of physical activity (81.94%), non-smoking (70.47%), blood pressure (75.2%), blood glucose (86.95%), and cholesterol indicators (74.49%). There was a higher percentage than in the ever infertile population. A greater proportion of participants who had been infertile were in the poor categories for body mass index (51.49%) and dietary intake (51.82%). Compared to the fertile group, the ever infertile group had a higher proportion of smokers, those with elevated blood pressure, blood glucose, cholesterol levels and body mass index.

### 3.2 Association between LS7 and fertility status

**Table 3** shows the results of the multivariate regression analysis. In the unadjusted model, the OR for glucose score was 0.59 (95% CI: 0.48-0.71), indicating a significant association with the risk of infertility. Upon controlling for age, education level, and race variables, the observed negative correlation remained statistically significant in model 2 [0.68 (0.55-0.85)]. The findings from Model 3, which was fully adjusted, indicate that there is a negative association between an increase of glucose score and the risk of infertility. Specifically, the OR was 0.64, with a 95% CI ranging from 0.51 to 0.80, suggesting an 37% decrease in the risk of infertility.

**Table 3:** The association between Life’s Simple 7 components score and infertility.

	Model 1	Model 2	Model 3
	OR (95% CI)	OR (95% CI)	OR (95% CI)
	*P-value*	*P-value*	*P-value*
Physical activity score	0.99 (0.68-1.46)	1.13 (0.74-1.72)	1.11 (0.70-1.75)
	0.980	0.665	0.718
Smoking score	0.84 (0.73-0.96)	0.90 (0.76-1.07)	0.83 (0.68-1.01)
	0.030	0.308	0.126
Blood pressure score	0.72 (0.57-0.91)	0.91 (0.70-1.19)	0.83 (0.63-1.10)
	0.030	0.605	0.270
Body mass index score	0.72 (0.59-0.88)	0.77 (0.62-0.96)	0.75 (0.60-0.94)
	0.008	0.047	0.047
Glucose score	0.59 (0.48-0.71)	0.68 (0.55-0.85)	0.64 (0.51-0.80)
	<0.001	0.007	<0.001
Cholesterol score	0.70 (0.56-0.87)	0.82 (0.64-1.04)	0.83 (0.64-1.07)
	0.008	0.191	0.242
Dietary intake score	0.99 (0.79-1.24)	0.89 (0.71-1.12)	0.85 (0.67-1.07)
	0.977	0.407	0.242

Model 1: no covariates were adjusted.

Model 2: age, education level and race were adjusted.

Model 3: age, educational level, race, poverty ratio and marital status were adjusted.

OR, odds ratios (95% CI) 95% confidence intervals.

The Benjamini-Hochberg method was used to adjust p values for multiple testing.

**Table 4** showed that the OR for LS7 was 0.88 (95% CI: 0.83-0.94), indicating a significant association with the risk of infertility. Upon controlling for age, education level, and race variables, the observed negative correlation was statistically significant in model 2 [0.92 (0.86-0.98)]. The findings from Model 3 suggested that there is a negative association between an increase of 1 unit in LS7 metrics and the risk of infertility. Specifically, the OR was 0.89, with a 95% CI ranging from 0.83 to 0.95, suggesting an 11% decrease in the risk of infertility. Participants who achieved ideal scores on LS7 metrics exhibited a reduced risk of infertility when these metrics were considered as a categorical variable. Similarly, the group with ideal scores had a significantly lower OR for infertility than those with poor scores. Comparable findings were also noted in model 2. After controlling for all covariates, the ideal group exhibited a 42% lower risk of infertility (OR = 0.42, 95% CI: 0.26-0.69) in comparison to the poor group.

**Table 4:** Association between Life’s Simple 7 score and infertility.

	Model 1	Model 2	Model 3
	OR (95% CI)	OR (95% CI)	OR (95% CI)
	*P-value*	*P-value*	*P-value*
Continuous			
Life’s Simple 7 score	0.88 (0.83-0.94)	0.92 (0.86-0.98)	0.89 (0.83-0.95)
	<0.001	0.015	0.004
Categorical			
Poor	ref	ref	ref
Intermediate	0.83 (0.61-1.12)	0.92 (0.68-1.26)	0.81 (0.57-1.13)
	0.248	0.600	0.248
Ideal	0.41 (0.26-0.64)	0.48 (0.30-0.75)	0.42 (0.26-0.69)
	<0.001	0.004	0.003

Model 1: no covariates were adjusted.

Model 2: age, education level and race were adjusted.

Model 3: age, educational level, race, poverty ratio and marital status were adjusted.

OR, odds ratios (95% CI) 95% confidence intervals.

Life’s Simple 7 score (0-14) Poor: (0-7); Intermediate: (8-10); Ideal: (11-14).

The Benjamini-Hochberg method was used to adjust p values for multiple testing.

### 3.3 Subgroup analyses

In the subgroup analyses, the observed interaction suggested that the influence of age on the relationship between LS7 and infertility is not consistent across different age groups (*P* for interaction < 0.001). Among individuals aged 35 or younger, each unit increase in LS7 score was associated with a substantial 18% (OR=0.82 [0.76-0.89]) decrease in the odds of infertility. However, in women older than 35 years, this association did not reach statistical significance in fully adjusted model. Similarly, subgroups stratified by the poverty level index were used to evaluate the association between the LS7 and the eternally infertile group. Using the fertile group as the reference group, the results revealed a substantial negative connection in fully adjusted model (**Table 5**). Using marital status as a subgroup for analysis, the relationship between LS7 and the risk of infertility was negatively associated only in those who were married and living with partner.

**Table 5:** The association between Life’s Simple 7 and infertility stratified by age, poverty level index and marital status.

	OR (95% CI)	*P-value*	*P for* *interaction*
Age			<0.001
<=35	0.82 (0.76-0.89)	<0.001	
>35	0.93 (0.85-1.02)	0.147	
Poverty level index			0.513
<=1.3	0.86 (0.78-0.95)	0.012	
1.3-1.85	0.83 (0.69-0.98)	0.048	
>1.85	0.90 (0.82-0.99)	0.048	
Marital status			0.240
Married	0.87 (0.79-0.96)	0.024	
Divorced/Separated/Widowed	1.00 (0.83-1.20)	0.970	
Never married	0.90 (0.81-1.00)	0.090	
Living with partner	0.78 (0.67-0.91)	0.038	

Analyses were adjusted for covariates age, educational level, race, poverty ratio, and marital status when they were not the strata variables.

OR: odds ratios (95% CI) 95% confidence intervals.

The Benjamini-Hochberg method was used to adjust p values for multiple testing.

## 4 Discussion

Our study is the first to explore the relationship between the American Heart Association’s LS7 metrics and fertility status between fertile and ever-infertile subjects in a U.S. population-based sample of women aged 20 to 44. The weighted study found that infertility was prevalent among women aged 18 to 44 at 17.66%, which aligns with the anticipated nationwide prevalence of 12 to 18% (28). The main finding of the study indicates a positive correlation between fertility and the LS7 Score, which reflects adherence to the American Heart Association’s LS7 metrics. This suggests that individuals who adhere more closely to these metrics are less likely to experience infertility. Notably, the odds of infertility decrease significantly as ideal cardiovascular health levels increase. This association remains significant even after adjusting for potential confounding factors. The reference group used in the study was individuals with poor LS7 metrics, chosen to highlight the importance of addressing unhealthy lifestyles. Importantly, the results emphasize the need to prioritize LS7 metrics in the general population, not only for reducing the risk of cardiovascular disease but also for mitigating the risk of infertility.

This study represents the inaugural attempt to examine the comprehensive array of ideal cardiovascular health metrics concerning infertility. Previous research has documented correlations between specific indicators of the LS7 framework, such as BMI (27), smoking (26), and dietary fiber (29)with self-reported infertility. Previous reports also observed older age (30), race (31, 32), and poverty (33) to be associated with higher odds of infertility. The fertility of individuals can be negatively impacted by various lifestyle and environmental factors, including but not limited to smoking and obesity (2). For instance, smoking has been associated with a reduced sperm count and quality in men, as well as an increased risk of infertility in women (34); excessive alcohol intake and caffeine consumption have been linked to infertility in both men and women (35); obesity, particularly in women, has been associated with an increased risk of infertility (36). However, being underweight can also negatively impact fertility in women; a diet high in trans fats, refined carbohydrates, and added sugars can negatively affect fertility in women, while a diet rich in dietary fiber, omega-3 fatty acids, and plant-based protein has been associated with improved fertility (37). Besides, some vitamins and minerals, such as folic acid, B12, and omega-3 fatty acids, have been suggested to improve fertility in women. Improving lifestyle factors can potentially improve fertility outcomes in patients with conditions like endometriosis and PCOS, which are associated with both cardiovascular disease and infertility. However, more research is needed to evaluate the potential benefits of managing cardiovascular risks using the LS7 metrics in patients with these conditions on their fertility outcomes (35).

The previous investigation indicated an elevated susceptibility to cardiovascular morbidity in subfertile women, albeit with a restricted sample size consisting solely of women who ultimately achieved childbirth (38). Previous studies have reported that women with endometriosis are at an increased risk of experiencing coronary heart disease later in life (39). The mechanisms that could explain the elevated risk of CVD in patients with endometriosis include inflammation, oxidative stress, and endothelial dysfunction (40). More studies of the cardiovascular-endometriosis interaction are needed to fully understand the underlying pathophysiology, possible means of early diagnosis, and prevention. Endometriosis is also associated with infertility, and the pathophysiology of both conditions shares some common biological pathways (40). Once again, it is important to note that these studies possess certain limitations regarding their scope, as they do not encompass cases of infertility that are idiopathic or undiagnosed.

The study conducted in LS7 found a correlation between smoking and infertility. The results indicated that individuals who had experienced infertility at any point in their lives had a noticeably higher prevalence of smoking compared to those who were fertile. According to a committee opinion, there is substantial evidence supporting a correlation between cigarette smoking and infertility. Researchers have identified several known toxins in the ovary and follicular fluid of individuals who smoke cigarettes. There exists a correlation between smoking and a shortened duration of the menstrual cycle, specifically those lasting 24 days or less. This association has been observed to potentially lead to a decrease in fertility (41). The present study has identified correlations between body mass index and infertility. The BMI is a fundamental metric for assessing obesity. Consistent with prior research, there is evidence indicating that obese women experience impaired stromal decidualization. This phenomenon could potentially elucidate the causes of infertility resulting from compromised receptivity and subsequently contribute to the development of placental abnormalities. In addition, it has been observed that women who are obese are at a higher risk of experiencing ovulatory dysfunction as a result of the dysregulation of the hypothalamic-pituitary-ovarian axis (42, 43). PCOS is a prominent etiological factor contributing to infertility. The development of PCOS frequently involves the occurrence of insulin resistance, which subsequently gives rise to various cardiometabolic abnormalities such as dyslipidemia, hypertension, glucose intolerance, diabetes, and metabolic syndrome (MetS). Consequently, women with PCOS face an elevated susceptibility to cardiovascular disease (44). Consistently, our results also implied a negative association between an increase of glucose score and the risk of infertility. Observational evidence has indicated a shared etiology between impaired glucose tolerance, cardiovascular risk, and fertility problems. Elevated sugar levels have been associated with pregnancy complications, and poorly controlled sugar levels may lead to an increased risk of infertility and miscarriage (45). The results indicate a convergence in the underlying mechanisms that contribute to both infertility and cardiovascular disease, which are complex multifactorial syndromes (9). For instance, the activation of the hypothalamic-pituitary-adrenal (HPA) axis has been implicated in the pathogenesis of both conditions (46, 47). The activation of neuroendocrine pathways is associated with stress, which has been independently linked to the development of MetS, cardiovascular disease, and infertility (48). Therefore, infertility may serve as an indicator of cardiometabolic disorders that may be initiated by neuroendocrine or other common mechanisms, and that could potentially be mitigated through timely intervention. An additional research investigation revealed that the adverse cardiovascular characteristics observed in women with PCOS during their reproductive years could potentially impact the well-being of their offspring, in addition to being influenced by genetic factors (49). The primary practical implication of our study is that adhering to the American Heart Association’s LS7 cardiovascular health metrics may play a crucial role in the prevention of infertility. Ideally, implement a multifactorial intervention targeting the key LS7 metrics to establish a more comprehensive understanding of the subject matter. The LS7 tool is a straightforward and economically efficient instrument for assessing CVH, with potential applications in monitoring and advancing a novel approach centered on fertility health. The LS7 metrics frequently exhibit interdependent interactions that can collectively contribute to the manifestation of infertility. The utilization of a composite score has the potential to offer valuable insights into the prediction of infertility rates. However, further research should be conducted in the form of prospective studies to substantiate and validate this claim. One of the important ways of clinical treatment for infertility continues to be lifestyle modifications. Given this consideration, when women seek medical assistance for infertility, healthcare providers are presented with a distinctive opportunity to offer guidance to women in their reproductive years regarding behavioral modifications that could potentially reduce the likelihood of developing chronic diseases in the future. It is crucial to address these matters while women are still capable of implementing such changes (50).

According to the present studies, although several studies have suggested an association between endometriosis, cardiovascular disease, and infertility. Women with infertility, particularly related to ovulation disorders and endometriosis, may be at an increased risk of experiencing coronary heart disease later in life (39). Additionally, endometriosis is associated with a higher risk of cardiovascular outcomes, potentially due to factors such as chronic inflammation, oxidative stress, and atherogenic lipid profile (51, 52). As for the impact of controlling cardiovascular risks on fertility outcomes in patients with endometriosis, the research is still limited. However, given the potential interplay between endometriosis, CVD, and infertility, managing cardiovascular risks in these patients could be beneficial for both cardiovascular health and fertility outcomes. Further research is needed to fully understand the underlying mechanisms and the potential benefits of managing cardiovascular risks in patients with endometriosis on their fertility outcomes.

The clinical significance of the observed association between LS7 scores and infertility in our study is underscored by the implications for reproductive health and cardiovascular well-being. Our findings reveal a noteworthy 58% reduction in the odds of infertility among individuals with ideal LS7 scores compared to those with poor scores. From a clinical standpoint, these findings have several noteworthy implications. Firstly, they emphasize the interconnectedness of cardiovascular health and reproductive function (5, 9, 39), supporting the concept of a shared pathophysiological basis. Secondly, the observed reduction in infertility odds among individuals with ideal LS7 scores implies that interventions aimed at improving cardiovascular health may potentially exert a positive influence on fertility outcomes (2, 26, 35–37). Furthermore, the identification of modifiable risk factors within the LS7 framework provides clinicians with actionable targets for intervention and risk reduction. Strategies aimed at optimizing lifestyle factors encompassed by the LS7, such as smoking cessation, regular physical activity, and a heart-healthy diet, may not only contribute to cardiovascular health but also hold promise in the realm of reproductive medicine. In conclusion, the observed association between LS7 scores and infertility suggests a potential avenue for preventive interventions that target cardiovascular health, offering a nuanced perspective on the broader implications of maintaining ideal cardiovascular well-being for reproductive outcomes.

This study utilized data from the NHANES, a comprehensive dataset obtained through population-based sampling techniques that were implemented consistently throughout the United States. The study samples exhibited greater representativeness due to the inclusion of appropriate NHANES sampling weights in all analyses. However, there are certain limitations on our study. Firstly, the self-report measure of infertility has certain limitations. Specifically, women may experience difficulties in accurately recalling the duration of their attempts to conceive, leading to potential misclassification of the length of time dedicated to conception efforts. Similarly, women clinically diagnosed with infertility prior to trying to conceive for 12 months or who had not tried to conceive may not have been included in our measure of infertility. This category encompasses women who have been diagnosed with endometriosis or PCOS, as well as women who are above the age of 35 and have been unable to conceive after six months of unsuccessful attempts. The underlying mechanism still needs to be determined. Secondly, due to the cross-sectional character of our research, we were unable to establish a causal link between LS7 and infertility. Thirdly, multiple other factors such as occupational exposure and genetic variants may also contribute to the pathology of infertility, further study needs to be done in this research field. Fourthly, the group with infertility may vary, and the LS7 may change with age, these confounding factors may lead to bias to some extent. Lastly, our study lacks specific data on PCOS, limiting our ability to discern whether the observed association between LS7 scores and infertility is independent of PCOS. While we acknowledge the potential relevance of PCOS, our focus was on the general relationship between LS7 scores and infertility, without specific subgroup analyses for PCOS. This study calls attention to the need for targeted research exploring nuanced associations between LS7 scores, infertility, and specific conditions such as PCOS.

## 5 Conclusion

In the population of women aged 18-44 in the United States, our research suggests a correlation between higher scores on the LS7 metric and a reduced likelihood of experiencing infertility. Additional research is warranted as LS7 metrics represent an ideal state of cardiovascular health, serving as a reliable indicator of a healthy lifestyle. Moreover, these metrics hold potential as a novel approach to addressing infertility concerns. The results of this study provide a new insight that the measurements of preventing cardiovascular diseases may also be associated with a lower prevalence of infertility.

## Data availability statement

Publicly available datasets were analyzed in this study. This data can be found here: www.cdc.gov/nchs/nhanes/.

## Ethics statement

The studies involving humans were approved by NCHS Ethics Review Board (ERB). The studies were conducted in accordance with the local legislation and institutional requirements. Written informed consent for participation was not required from the participants or the participants’ legal guardians/next of kin in accordance with the national legislation and institutional requirements. Written informed consent was obtained from the individual(s) for the publication of any potentially identifiable images or data included in this article.

## Author contributions

LW: Conceptualization, Data curation, Formal Analysis, Methodology, Writing – original draft, Writing – review & editing. GC: Data curation, Formal Analysis, Methodology, Writing – original draft. SC: Conceptualization, Supervision, Writing – original draft, Writing – review & editing. XZ: Data curation, Formal Analysis, Methodology, Writing – review & editing. MQ: Data curation, Formal Analysis, Methodology, Writing – original draft. YT: Data curation, Formal Analysis, Methodology, Writing – review & editing.

## Appendix

**Appendix 1:** Definition of Life’s Simple 7 Cardiovascular Health Metrics.

Metric	Life’s Simple 7 Cardiovascular Health		
	Poor	Intermediate	Ideal
Blood pressure	Treated BP≥140/90 mm Hg, and BP ≥140/90 mm Hg	SBP 120-139 mm Hg or DBP 80-89 mm Hg or treated to <120/80 mm Hg	<120/80 mm Hg, without BP-lowering meds
Cholesterol	≥240 mg/dL	200-239 mg/dL or treated to <200 mg/dL	<200 mg/dL, without lipid-lowering medication
Glucose	HbA1c >6.4%	HbA1c 5.7%-6.4% or treated with insulin or oral meds to HbA1C <5.7%	HbA1c <5.7%, without meds
Smoking	Current smoker	Former smoker	Never smoker
Body mass index	BMI≥30 kg/m²	25-29.9 kg/m²	<25 kg/m²
Physical activity	No activity	1-149 minutes moderate/vigorous per week	≥150 minutes moderate/vigorous per week
Dietary intake	HEI<50	HEI 50-80	HEI>80

AHA indicates American Heart Association; FPG, fasting plasma glucose; BMI, body mass index; BP, blood pressure; SBP, systolic blood pressure; DBP, diastolic blood pressure; HbA1C, hemoglobin A1c; HEI, Healthy Eating Index;

The AHA definitions for poor, intermediate, and ideal health were used for blood pressure, cholesterol, BMI, and physical activity; however, modified definitions were used for FPG, smoking, and health diet score.

Glucose, AHA defined poor health as FPG ≥126 mg/dL or HgbA1C ≥7%, intermediate health as FPG 100 to 125 mg/dL or HgbA1C <7%, and ideal health as FPG <100 mg/dL.

Smoking, AHA defined poor health as current smoker, intermediate health as quit smoking <12 months, and ideal health as never smoker or quit smoking ≥12 months.

HEI score includes 3 of 5 primary criteria included in the AHA healthy dietary score: fruits and vegetables, whole grains, and sodium. Not included are sugar-sweetened beverages and fish consumption.
